# Stress, Personality, Attachment, and Coping Strategies During the COVID-19 Pandemic: The STERACOVID Prospective Cohort Study Protocol

**DOI:** 10.3389/fpsyt.2022.918428

**Published:** 2022-06-27

**Authors:** Arlette Edjolo, Jean-Michel Dorey, Mathieu Herrmann, Catherine Perrot, Cécile Lebrun-Givois, Aurélie Buisson, Hanane El Haouari, Bernard Laurent, Elodie Pongan, Isabelle Rouch

**Affiliations:** ^1^CROMA, University Hospital of Saint-Etienne, Saint-Etienne, France; ^2^INSERM, U1219, Bordeaux Population Health Center, University of Bordeaux, Bordeaux, France; ^3^Department of Aging Psychiatry, Hospital Le Vinatier, Bron, France; ^4^Memory Clinical and Research Center of Saint Etienne (CMRR), Geriatric Unit, University Hospital of Saint-Etienne, Saint-Etienne, France; ^5^INSERM, U1028, Neuropain Team, Lyon Neuroscience Research Center, Lyon, France

**Keywords:** COVID-19 pandemic, lockdown, older adults, psychiatric disorders, PTSD, personality, attachment, coping strategies

## Abstract

**Background:**

Due to the lockdown linked to the COVID-19 pandemic, the French National Authority for Health has recommended reinforced follow-up of psychiatric patients, with particular attention to people over 65 years. Cross-sectional studies reported an increased risk of anxiety, depression, and suicide during this period. Older people with psychiatric disorders are at higher risk of developing severe COVID-19 and worsening their psychiatric symptoms.

**Objective:**

The main objective is to evaluate the link between coping strategies and the onset of post-traumatic stress disorder (PTSD) after lockdown. The secondary objective is to assess the psychological factors influencing lockdown experiences such as personality, attachment type, or coping strategies.

**Method/Design:**

this is a multicenter cohort study including 117 patients followed up by phone in two French geriatric psychiatry units. Sociodemographic variables, psychiatric diagnoses, lockdown conditions, coping strategies, anxiety, and depressive symptoms reported during the first lockdown will be collected retrospectively from the medical file. A first prospective assessment including personality traits, attachment type, and traumatic life events will be conducted at 12 months (T1). Follow-up visits assessing anxious-depressive symptoms and PTSD will be made 18 (T2) and 24 months (T3) after the first lockdown. The primary outcome measure is PTSD symptoms. Secondary outcomes measures are coping strategies, generalized anxiety, anxiety about the COVID-19 pandemic, and quality of life.

**Discussion:**

This study aims to determine if the type of coping strategies usually employed have an impact on the onset of PTSD after a lockdown period. It will also determine if these coping strategies are influenced by other factors such as sociodemographic variables, lockdown conditions, particular personality traits, attachment type, and traumatic life events. This study could help identify factors associated with a poorer experience of lockdowns and pandemic crisis in elderly patients followed in a psychiatric center, and guide support in future similar situations.

**Trial Registration:**

ClinicalTrials.gov: http://clinicaltrials.gov/show/NCT04760795, Registered 18 February 2021.

## Introduction

In December 2019, the World Health Organization (WHO) (1) reported a new coronavirus (SARS-CoV-2) that quickly became pandemic, leading worldwide countries to take drastic measures. From March 2020, in France (2) unprecedented measures have been voted to limit travel (3), prescribing social distancing and isolation. Thus, several factors could affect the population's mental health, including the lack of scientific data on coronavirus disease 2019 (COVID-19), 24-h news channels reporting daily death count, and the government's lack of visibility on lockdown measures.

The epidemic situation and the resulting measures being unprecedented, the psychological consequences are difficult to predict. Previous studies on the mental health of the population, on the impact of quarantine measures, have been carried out on epidemics such as the severe acute respiratory syndrome (SARS) in 2003, the H1N1 flu in 2009 and 2010, the Middle East Respiratory Syndrome (MERS) in 2012, or Ebola in 2014. A recent literature review showed that quarantine measures hurt mental health leading to Post-Traumatic Stress Disorder (PTSD) susceptible to last for months or even years after measures implementation [Brook et al., (4)]. Indeed, anxiety, depressive symptoms, and PTSD were reported in the general population more than 1 year after the Ebola epidemic in 2014 (5). Some factors might affect the negative impact of quarantine, among them: sociodemographic characteristics and lockdown conditions (6), insufficient access to necessities including medical supplies and regular medical care (7–9), a lack of clarity in the information provided by the public health authorities, and a quarantine period exceeding 10 days (10). At the beginning of 2020, the French population experienced issues with the supply of masks and a long lockdown duration. Additionally, factors such as the government's lack of visibility on lockdown measures and 24-h news channels reporting daily deaths count could have negatively affected the population's mental health.

Self-protective measures such as wearing a mask or social distancing could have increased the onset of some disorders such as compulsive hand washing and agoraphobia, as shown by previous studies (11). Other long-term effects could be observed. Regarding the current COVID-19 pandemic, previous cross-sectional studies showed an increase in anxiety symptoms and depression, higher alcohol consumption, or poorer psychological wellbeing (12, 13). An international population-based study (14) also reported that the lockdown experience was linked to higher risks of depression, PTSD, and suicide (15).

In this context, the elderly population with psychiatric disorders, who is already frailer, might have poorer psychic health. Indeed, from the beginning of the pandemic, older people were more susceptible to developing severe COVID-19 (16). In addition, the media underlined the fragility of this age group and speculated on the choices that could be made by the intensive care services to favor younger patients in case of saturation. Next, older adults being less familiar with recent technologies and less prone to use video screen communications, the limitation of outings worsened their social isolation. The lockdown also complicated their daily organization because they usually depend on their children or external help (17). Finally, they also experienced more financial precariousness, knowing that socio-economic conditions have been identified as a risk factor for psychological disorders after quarantine measures during previous epidemics (10).

For patients with psychiatric disorders, the risks of social isolation and financial precariousness are increased, as is the risk of medical comorbidities and particularly cardiovascular comorbidities (18), making them even more vulnerable to the virus. Moreover, the onset of PTSD is more frequent in a population with a prevalent psychiatric pathology (19); and a longitudinal study (20) showed that psychiatric history induced anger and anxiety symptoms several months after the end of quarantine. Finally, psychiatric history increased the risk of suicidal ideation during the COVID-19 pandemic (14).

The concept of “coping” was defined by the set of cognitive and behavioral efforts put in place to control, reduce or tolerate internal and external demands that threaten or overwhelm an individual's resources” (21). Two types of coping were distinguished: problem-focused coping which constitutes the efforts undertaken to confront the situation, and emotion-focused coping which aims to decrease the emotional distress related to the situation. A synthesis of studies carried out on the adjustment strategies reveals the third set of strategies: seeking social support (22). Indeed, according to the situation, social support could help to change the problem or emotional state.

Bruchon-Schweitzer identified two determinants of the coping type: dispositional factors (personality traits, beliefs, motivation) and contextual factors, related to the context (nature of the situation, its imminence, duration, ambiguity, frequency, intensity, and controllability). Certain personality traits from the “Big Five” model (23) might influence the coping type. Neuroticism is defined as emotional instability and a persistent trend of negative emotions and might be more often associated with emotion-focused strategies (24).

An individual's response to stress might be associated with the quality of relationships, particularly the attachment dimensions. On the one hand, the attachment type seems to condition the physiological response to stress and the level of stress perceived by individuals (25). On the other hand, the way to face stress is related to attachment: Ognibene and Collin (26) studied the coping strategies employed according to attachment type in young adults. They showed that individuals with a secure attachment type received more help from friends and family and sought more social support when facing a stressful situation. While individuals with a preoccupied attachment type sought social support and tended to use avoidance strategies. Individuals with fearful and dismissing attachment types were less prone to seek social support.

Previous studies on traumatic events showed that some strategies, particularly problem-focused and seeking social support, were more efficient to prevent psychological repercussions such as PTSD (27, 28).

To our knowledge, no study has investigated coping strategies in epidemic or lockdown situations.

The French Health Authority has recommended a follow-up of the older psychiatric patients during the lockdown period. It advocated maintaining and reinforcement of outpatient care services by favoring teleconsultation by video or phone (2).

The present study includes a population of older outpatients with psychiatric disorders followed-up by phone in French hospitals over the lockdown period.

According to previous studies, we think that: a/ Patients could experience severe anxiety symptoms during this period. This anxiety might last after lifting the lockdown restrictions. However, we hypothesize that individuals who implement problem-focused and social support-seeking coping strategies might be less anxious. Additionally, the type of coping strategies used could influence the onset of PTSD; b/ Factors such as lockdown conditions, personality traits, attachment type, and life events might affect the coping strategies.

The primary aim of the study is to investigate the link between coping strategies and the onset of PTSD after lockdown.

The secondary objectives are threefold: a/Identify the coping strategies used during the COVID-19 crisis and measure their efficiency on generalized anxiety and anxiety about the COVID-19 pandemic, b/Assess the factors which influence the coping strategies used usually and during the lockdown, especially the sociodemographic variables, the lockdown conditions, the type of personality, the attachment type, and the presence of traumatic life events, c/ Evaluate the impact of PTSD on quality of life.

## Methods and Analysis

### Study Design

**Figure 1:**
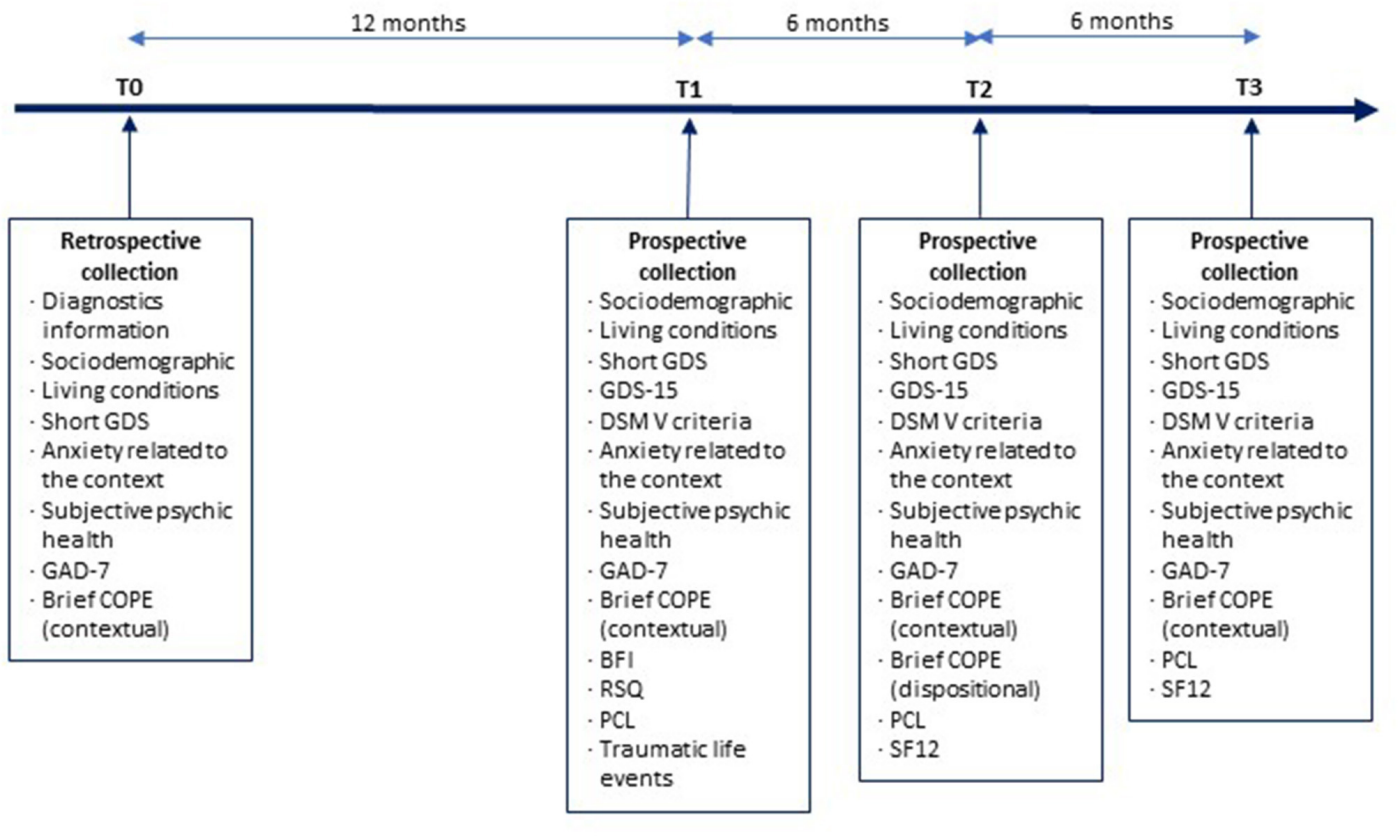
The typical schedule for a patient enrolled in the STERACOVID protocol: a summary of the distinct stages.

It is a bicentric observational prospective cohort study. Figure 1 displays the typical schedule for a patient enrolled in the STERACOVID protocol.

### Setting and Patients

The STERACOVID study participants will be recruited among outpatients followed up by phone for psychiatric diseases during the lockdown related to COVID-19 in the geriatrics department of the University Hospital of Saint-Etienne and the aging psychiatric department of the Vinatier Hospital, Lyon, France.

#### Inclusion Criteria

Patients must: (1) be outpatient with a psychiatric pathology; (2) be 60+; (3) regularly followed in the psychiatric unit; (4) be followed by teleconsultation during the first lockdown due to COVID-19; (5) be affiliated or entitled to a social security scheme.

#### Exclusion Criteria

Patients with major neurocognitive disorders or hospitalized were excluded from the study.

### Study Withdrawals

Patients withdraw from the study in case of refusal or inability to continue the study for medical reasons. They will not be replaced. Patients will be followed-up as part of their usual care with no substitution treatment.

### Independent Variables

Independent variables will be:

- Sociodemographic characteristics will be collected: Age in years, sex (male/female), and marital status (single/in couple/widowed/divorced). For education, low and high educational levels are defined by the duration of schooling, with a threshold of 12 years (≥12 years vs. <12 years).
- Information on lockdown period: total lockdown without outings (yes/no); social network (maintaining family contact, professional help visits); isolation (does the patient live alone at home?); feeling of loneliness (never/sometimes/often/very often); adoption of protective measures (yes/no); diagnosis of COVID-19 for the patient and/or in the close circle (yes/no); bereavement due to COVID-19; feeling about vaccine (willing to get vaccinated (yes/no/doesn't know/already vaccinated), reception of the announcement of a vaccine (reassured/indifferent/other)); obsessive thoughts of contamination (yes/no); presence and frequency of episodes of panic attack; worry (about oneself, relatives and/or the future); suicidal ideations (yes/no); delusional syndrome eventually linked to the lockdown (yes/no); the past time in watching TV news (0 h/ <1 h/1–3 h/more than 3 h), presence of daily routine (yes/no); information on tobacco and alcohol consumption, and sleep disorders (if yes, increased/stable/decreased), information on pain (type, duration, onset, daily interference, daily analgesic medication).
- The personality will be assessed using the Big Five Inventory (BFI) (29). Built-in English, this self-report inventory has been validated in French and includes 45 items designed to measure the 5 dimensions of personality: openness (open-mindedness, originality), conscientiousness (control, goal-directed behaviors), extraversion (energy, enthusiasm), agreeableness (affection, altruism), and neuroticism (negative mood, anxiety). Each item consists of a short phrase and is rated on a 5-point Likert scale (1 = *disagree a lot* to 5 = *agree a lot* Scale scores are the sum of the items for each subscale.
- The attachment type will be evaluated using the Relationship Scales Questionnaire (RSQ) (30). It is a self-reported multidimensional questionnaire including 30 items among which 17 items are specific to relationships and resulting in 4 subscales defining 4 attachment prototypes:

- The “secure” subscale (S) consists of 5 items. The “secure” attachment type indicates that the individual has positive views of self and others. Others are generally assumed available and helpful if necessary.
- The “fearful” subscale (F) consists of 4 items. It indicates negative perceptions of self and others. Others are generally assumed not available.
- The “preoccupied” (P) subscale consists of 4 items. It indicates a negative view of self and a positive view of others. The individual has a sense of personal worthlessness in the eyes of others and the belief that others are not available or caring in case of need.
- The “dismissing” (D) subscale consists of 5 items. It relates to a high level of self-confidence and negative views of others. The individual has a sense of self-worth that one owes only to oneself and very negative expectations of others from whom one should, on the other hand, expect nothing.

Each item is rated on a 5-point Likert scale (1 = *not at all like me* to 5 = *very much like me*). The four combining scores allow calculating the scores of two latent dimensions: the “self-model” and the “others” model.

- The presence of traumatic life events will be collected. Patients will be asked if they experienced one or several traumatic life events.

### Dependent Variables

- The primary endpoint will be PTSD, assessed using the original version of the PTSD checklist (PCL) (31). It focuses on symptoms related to a single traumatic event. The PCL is a standardized self-reported rating scale including 17 items assessing the intensity, in the last month, of 17 PTSD key symptoms. Each item uses a 5-point Likert scale ranging (from 1 = *not at all* to 5 = *extremely*). Scores consist of the total symptom severity (sum of all items going from 5 to 85) and scores of three subscales corresponding to 3 for three PTSD sub-syndromes: re-experiencing of the traumatic event, avoidance of trauma reminders, and hyperarousal.

The secondary endpoints will be:

- The coping strategies usually employed will be assessed using the Brief Coping Orientation to Problems Experienced (COPE) Inventory (32). This scale is a short version of the COPE Inventory developed by Caver and has been validated for a French population. This multidimensional instrument has 14 subscales composed of 2 items each: (1) active coping, (2) planning, (3) seeking instrumental social support, (4) seeking emotional social support, (5) focus and venting emotions (wanting to express feelings), (6) behavioral disengagement, (7) mental disengagement (distraction), (8) self-blaming, (9) positive reinterpretation, (10) humor, (11) denial, (12) acceptance, (13) turning to religion, and (14) substance use. According to guidelines, the scale might be used from a dispositional or contextual perspective. In this study, the contextual (pandemic situation) and dispositional versions will be used.
- Anxiety in the last 2 weeks will be assessed during phone medical interviews using the Generalized Anxiety Disorder scale (GAD7). The scale was validated in French (33). It consists of 7 items: (1) feeling nervous, anxious, or on edge, (2) not being able to stop or control worrying, (3) worrying too much about different things, (4) having trouble relaxing, (5) being so restless that it is hard to sit still, (6) becoming easily annoyed or irritable, and (7) feeling afraid as if something awful might happen. Each item is 4-scaled: 0- never; 1- several days; 2- more than half the days; 3- nearly every day.
- Anxiety about the COVID-19 crisis will be assessed during medical phone visits using a 10-point ordinal scale (from 0: free of stress to 10: maximum of stress) by answering the question: “On a scale from 0 to 10, how much stress do you feel in the context of COVID-19?”
- Subjective psychic health will be assessed during medical phone visits using a 10-point ordinal scale (from 0: the worst to 10: the best) by answering the question: “How do you rate your mental health? (From 0, the worst ever to 10, the best).”
- Quality of life will be assessed using the 12-Item Short-Form Health Survey (SF-12) (34), a short version of the Medical Outcomes Study Short-Form General Health Survey (SF36) (35). This self-reported questionnaire explores the quality of life related to physical, emotional, and social health. The 12 questions concern the four last weeks and investigate eight domains: physical activity, life and relationships with others, physical pain, subjective health, vitality (energy and fatigue), limitations related to the psychic status, physical condition, and psychic health. The SF-12 allows calculating a mental quality of life score and physical quality of life score.

### Data Collection Procedure

The data collection procedure is displayed in Figure 1 and Appendix (Table A).

#### Inclusion Visit and Retrospective Data Collection

Inclusion and exclusion criteria verification will be based on the patients' records. At T0, between March 17^th^ and May 10^th^, 2020, sociodemographic characteristics, psychiatric diagnoses, and lockdown conditions will be collected in medical records if available. Data will be anonymized and stored in a database. Anxiety (GAD-7) and coping scores (Brief COPE) will be collected for Saint Etienne participants during clinical visits and reported in the database.

#### Follow-Up

Lockdown assessment is proposed for participants 12 (T1), 18 (T2), and 24 months (T3) after the first lockdown.

The patients withdraw from the study at the end of their participation which will be for a maximum of 2 years.

### Bias Mitigation Measures

All consecutive patients followed up in phone visits who agree to participate in the research and meet the inclusion criteria will be included in the study to avoid selection bias.

Participants will be blinded to the study assumptions to avoid information bias. They will receive the explanations at the end of the session.

The evaluation criteria are sufficiently objective parameters to avoid any evaluation bias.

### Data Analysis

#### Sample Size

Given the clinical context of the study population, the expected prevalence of PTSD might be 30% in patients with emotion-focused coping strategies and 10% in those with problem-focused strategies. Assuming an α risk at 5% and power (1-β) of 80% (two-sided test), the number of required subjects would be 59 per group, i.e., 118 subjects in total.

#### Statistical Analysis

Descriptive analyses will be conducted using Student *t*-tests for quantitative variables and Chi-square tests for qualitative variables. Kruskal-Wallis tests will be performed for non-normally distributed variables. Logistic regression models will be performed to investigate the association of coping strategies with (1) PTSD, (2) personality traits, (3) attachment type, and (4) the presence of life traumatic events. Associations between coping strategies and anxiety will be performed using linear regression models. Univariate and multivariate models will be performed to consider potential confounding factors such as environmental, psychiatric, and socio-cultural factors. Moreover, structural equation modeling including psychometric instruments will be used when appropriate.

For all analyses, a *p* < 0.05 is considered statistically significant and the 95% confidence intervals (CI) will also be provided.

## Discussion

The STERACOVID study will be the first longitudinal study to investigate coping strategies in the COVID-19 pandemic context in the psychiatric geriatric population. This study aims to determine if the type of coping strategies used has an impact on the onset of PTSD after a lockdown period. It will also determine if these coping strategies are influenced by other factors such as socio-demographics, lockdown conditions, particular personality traits, attachment type, and traumatic life events. Several studies (4, 12–14) suggested that quarantine and pandemic crises hurt mental health leading to PTSD, anxiety, and depressive symptoms.

The originality relies on the choice of the study population and the longitudinal design going from the beginning of the pandemic over the different lockdown and post-lockdown periods. Moreover, it includes several standardized and validated scales to assess PTSD, depressive symptomatology, anxiety, personality trait, attachment type, and coping strategies. We also expect to limit the recruitment bias by using phone call interviews conducted by trained psychologists.

The STERACOVID study also has some limitations. Although most assessment tools are standardized and validated scales and constructs, anxiety about COVID-19, feeling of loneliness, and subjective psychic health assessments rely on unvalidated single-item questions. Thus, for these secondary endpoints, we will conduct correlation tests with validated scales such as the GAD7 scale or the psychic health part of the SF-12 scale.

To have a sufficiently large sample, we chose not to include the types of psychiatric diagnoses in the inclusion criteria. Additionally, this will better reflect the patients seen in medical practice. However, the diagnoses classified according to ICD 10 will be collected, and the analyses will take this information into account.

This study will allow identifying the coping strategies to develop in the psychiatric population of older adults and help clinicians track patients at risk to build better care support. It will favor the implementation of more relevant coping strategies, and better lockdown living conditions to limit anxiety and PTSD.

## Ethics Statement

The studies involving human participants were reviewed and approved by the Ethics Committee of the Saint-Etienne University Hospital. This committee covers the ethical approval for the three sites of our data collection. All procedures follow the Declaration of Helsinki and the International Conference on Harmonization (ICH) Good Clinical Practice Guidelines. The patients have received an informed written information notice. The STERACOVID study is registered in the Clinical Trials database (Current Controlled Trials NCT04760795 http://clinicaltrials.gov/show/NCT04760795). The Ethics Committee waived the requirement of written informed consent for participation.

## Author Contributions

IR conceived the idea for the study, helped to draft the manuscript, and managed the design and coordination of the study. EP participated in its design and coordination management. AE drafted the manuscript for submission to BMC Psychiatry. J-MD, AB, MH, BL, CP, and CL-G participated in its design and coordination. IR, AB, HE, CP, and CL-G are responsible for the participants' inclusion at the University Hospital of Saint-Etienne. J-MD and MH are responsible for the participants' inclusion at the Vinatier hospital. All authors approved the final version of the manuscript.

## Funding

The STERACOVID study is funded by the non-profit APICIL Foundation. The funds were allocated after the evaluation of several scientific experts and the deliberation of the scientific council of APICIL Foundation. Yet APICIL Foundation has no role or authority in the design or conduct of the study neither in the collection, analysis, interpretation of the data; preparation, review, or approval of the manuscript; or the decision to submit the manuscript for publication.

## Conflict of Interest

The authors declare that the research was conducted in the absence of any commercial or financial relationships that could be construed as a potential conflict of interest.

## Publisher's Note

All claims expressed in this article are solely those of the authors and do not necessarily represent those of their affiliated organizations, or those of the publisher, the editors and the reviewers. Any product that may be evaluated in this article, or claim that may be made by its manufacturer, is not guaranteed or endorsed by the publisher.

## Appendix

**Table A:** Summary of follow-up data collection.

	**T0**	**T1**	**T2**	**T3**
	**Retrospective collection**	**First prospective**	**Second prospective**	**Last prospective**
	**(on patient's records)**	**collection**	**collection**	**collection**
**Moment/Actions**	**Between March 17th and May 10** ^th^ **, 2020**	**T0 + 12 months**	**T0 + 18 months**	**T0 + 24 months**
Diagnostics information	X			
Sociodemographic	X	X	X	X
Interview on living conditions	X	X	X	X
Depression				
Short GDS	X	X	X	X
GDS 15			X	X
DSM V criteria			X	X
Anxiety about the context (ordinal scale)	X	X	X^*^	X^*^
Subjective psychic health (ordinal scale)	X	X	X	X
Generalized anxiety (GAD-7)	X	X	X	X
Contextual Coping strategies (BRIEF COPE)	X	X	X	X^*^
Dispositional Coping strategies (BRIEF COPE)			X^*^	
Personality (BFI)		X		
Attachment type (RSQ)		X		
Traumatic life events		X		
Post-traumatic stress (PCL)			X	X
Quality of life (SF12)			X	X

* *To ask only if the virus is still circulating. The STERACOVID study, 2020-2022, France*.
